# Five-year outcomes of transcatheter mitral valve replacement in patients with severe symptomatic mitral regurgitation: results from the Tendyne Expanded Clinical Study

**DOI:** 10.1093/eschf/xvag175

**Published:** 2026-06-27

**Authors:** David W M Muller, Alison Duncan, Paul Sorajja, Gry Dahle, Vasilis Babaliaros, Edith Lubos, Vinay Badhwar, Paolo Denti, Marvin H Eng, Paul Grayburn, Brian Bethea, Darren Walters, Georg Nickenig, Anna Sonia Petronio, Erwan Donal, Vinod H Thourani, Mubashir Mumtaz, Thomas Modine, Nicolas Dumonteil, Jean-Francois Obadia, Samir R Kapadia, Mayra E Guerrero, Ricardo De Medeiros, Robert McNutt, Hong Nie, Michael Chuang, Philipp Blanke, Lenard Conradi

**Affiliations:** Cardiology Department, St.Vincent’s Hospital Sydney, Victoria Street, Darlinghurst, NSW 2010, Australia; Cardiology Department, Royal Brompton Hospital, London, UK; Banner Heart Institute, Banner University Medical Center, Phoenix, AZ, USA; Department of Internal Medicine, Abbott Northwestern Hospital, Minneapolis, MN, USA; Department of Cardiothoracic and Vascular Surgery, Oslo University Hospital, Oslo, Norway; Department of Medicine, Emory University Hospital, Atlanta, GA, USA; Department of Internal Medicine, University Heart Center Hamburg, Hamburg, Germany; Department Cardiovascular & Thoracic Surgery, West Virginia University, Morgantown, WV, USA; Cardiac Surgery Department, San Raffaele University Hospital, Milan, Italy; Department of Internal Medicine, University of Nebraska Medical Center, NE, USA; Department of Internal Medicine, University of Texas Southwestern, Dallas, TX, USA; Department of Cardiac Surgery, MedStar Union Memorial, Baltimore, MD, USA; Cardiology Department, The Prince Charles Hospital, Brisbane, Australia; Department of Cardiology, University Hospital Bonn, Bonn, Germany; Cardiothoracic and Vascular Department, Azienda Ospedaliero Universitaria Pisana, Pisa, Italy; Cardiology Department, CHU de Rennes, LTSI INSERM 1099, université de Rennes, France; Department of Cardiovascular Surgery, Piedmont Heart Institute, Atlanta, GA, USA; Department of Cardiothoraci Surgery, University Pittsburgh Medical Center Pinnacle Health, Harrisburg, PA, USA; Department of Cardiovascular Surgery, CHRU de Lille, Lille, France; Cardiology Department, Groupe CardioVasculaire Interventionnel, Clinique Pasteur, Toulouse, France; Department of Adult Cardiac Surgery, Hôpital Cardiologique Louis Pradel, Lyon, France; Department of Cardiovascular Medicine, Cleveland Clinic Foundation, Cleveland, OH, USA; Department of Cardiovascular Medicine, Mayo Clinic, Rochester, MN, USA; Abbott, St.Paul, MN, USA; Abbott, St.Paul, MN, USA; Abbott, St.Paul, MN, USA; Department of Medicine, Beth Israel Deaconess Medical Center, Boston, MA, USA; Deprtment of Radiology, St Paul’s Hospital, Vancouver, BC, Canada; Department of Internal Medicine, University Heart Center Hamburg, Hamburg, Germany

**Keywords:** Mitral regurgitation, Mitral stenosis, Mitral valve replacement, Tendyne, Transcatheter mitral valve replacement

## Abstract

**Aims:**

Transcatheter mitral valve replacement (TMVR) with the Tendyne Mitral Valve System is a treatment option for patients with severe symptomatic mitral regurgitation (MR) unsuitable for conventional mitral valve surgery or transcatheter edge-to-edge repair (TEER). This study sought to evaluate the safety and effectiveness of TMVR through 5-year follow-up.

**Methods and Results:**

The Tendyne Expanded Clinical Study is a prospective, single-arm, multicentre study that enrolled patients between November 2014 and June 2020. The study enrolled 191 patients (mean age 74.1 ± 8.0 years, 62.8% male, 70.2% New York Heart Association Class (NYHA) class III/IV, 88.5% secondary MR), of whom 186 (97.4%) underwent TMVR with Tendyne. MR grade decreased from ≥3+ in 99.5% patients at baseline to grade ≤1+ in 95.3% at 5 years. In those patients surviving to 5 years (*n* = 49), durable symptomatic improvement was evidenced by 73.5% patients being in NYHA class I/II at 5 years. The Kansas City Cardiomyopathy Questionnaire overall summary score increased from 48.5 ± 22.5 points at baseline to 67.6 ± 21.5 points at 5 years. Serious adverse events that occurred through 5 years included life-threatening bleeding (11.5%), fatal bleeding (2.6%), renal insufficiency/failure (28.8%), endocarditis (7.3%), device thrombosis (5.8%), and new onset atrial fibrillation (13.1%). No structural device degeneration or device embolism occurred through 5 years.

**Conclusions:**

The Tendyne TMVR was effective at achieving immediate and sustainable elimination of MR, which was associated with symptomatic improvement and absence of structural valve degeneration in a high-risk cohort over 5 years. These findings support TMVR with the Tendyne System as an alternative for patients with appropriate mitral valve anatomy and symptomatic secondary MR unsuitable for mitral surgery or TEER.

## Graphical Abstract

**Graphical Abstract:**
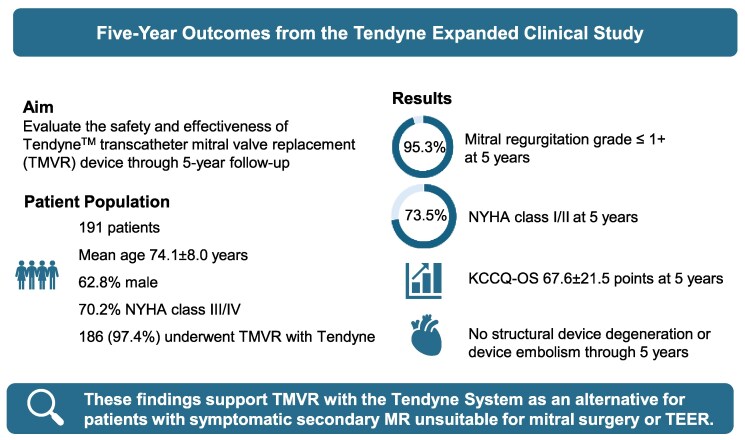

## Introduction

Severe, symptomatic mitral regurgitation (MR) affects millions of individuals worldwide and is associated with a high burden of morbidity and mortality.^1,2^ Although pharmacologic therapies may induce reverse remodelling and lessen MR severity,^3,4^ patients with persistent, severe MR face poor long-term outcomes.^5^

Current guidelines endorse surgical mitral valve repair or replacement as the gold-standard treatment for eligible patients,^6,7^ with transcatheter edge-to-edge repair (TEER) as a less invasive alternative for those at elevated surgical risk.^8^ Yet, a substantial subset of patients remain ineligible for either mitral surgery or TEER due to a combination of prohibitive operative risk, challenging anatomy, or comorbid conditions. Transcatheter mitral valve replacement (TMVR) thus remains an important treatment option for patients who are ineligible for mitral surgery or TEER, delivering predictable and complete elimination of MR.^9,10^

The Tendyne Mitral Valve System is a TMVR system that is delivered via a transapical approach without cardiopulmonary bypass. Initial 30-day and 3-year outcomes of the prospective Expanded Clinical Study demonstrated favourable safety and efficacy.^11–14^ In the current report, we detail the 5-year outcomes in patients enrolled in the Tendyne Expanded Clinical Study to define the long-term durability (symptom relief and structural valve degeneration) of TMVR, characterize late adverse events, and inform the role of this technology in the management of a cohort of high-risk patients with severe MR who were not suitable candidates for surgical replacement.

## Methods

### Study design and patients

The Tendyne Expanded Clinical Study (NCT02321514) is a single-arm, prospective, multicentre study. Details of the study device, design, procedure, and preceding follow-up outcomes have been described.^11–14^ Patients with MR grade ≥3+, functional impairment [New York Heart Association Class (NYHA) ≥II], and who were deemed unsuitable for surgery or TEER by the institution’s heart team were eligible. Key exclusion criteria included severe mitral annular calcification, severe mitral stenosis, left ventricular (LV) ejection fraction (LVEF) <30%, and LV end-diastolic diameter (LVEDD) >7.0 cm.

Eligible patients underwent transapical TMVR with the Tendyne device between November 2014 and June 2020 at 39 sites in Europe (France, Germany, Italy, Netherlands, Norway, Sweden, Switzerland, and UK), USA, and Australia. This study conformed with the principles outlined in the Declaration of Helsinki, with individual institutional review board or ethics committee approval at each site. All patients provided written informed consent. Patients who underwent an attempted procedure constituted the treated population, and those who left the operating room with the study device implanted constituted the implanted population.

Follow-up was performed through 5 years (pre-discharge, 1, 3, and 6 months, and 1 year and annually thereafter). Transoesophageal echocardiography (TEE) was performed intraoperatively, and transthoracic echocardiography (TTE) was used at all follow-up visits. All imaging was assessed by independent echocardiography and cardiac computed tomography (CT) core labs (Beth Israel Deaconess Medical Center, Boston, MA, USA and St. Paul’s Hospital, Vancouver, BC, Canada, respectively). Functional status was measured by the NYHA functional class and quality of life by the Kansas City Cardiomyopathy Questionnaire overall summary (KCCQ-OS) score. All cardiovascular and non-cardiovascular medications were documented at baseline and at each follow-up visit. Cardiovascular medications included anticoagulants, antiplatelet agents, ACE inhibitors, angiotensin II receptor blockers or inhibitors, beta blockers, calcium channel blockers, diuretics, digitalis preparations, and vasodilators.

### Statistical analysis

Categorical variables are reported as the number and percentage of observed data. Continuous data are reported as mean ± standard deviation unless otherwise stated. All-cause mortality is reported using Kaplan–Meier estimates. Comparisons between baseline and follow-up parameters were made using Exact McNemar’s test for categorical variables and Poisson Regression for count data (e.g. annualized heart failure hospitalization [HFH] rate) and repeated measure mixed model ANCOVA for continuous variables. Predictor analysis was performed using univariable and multivariable Cox proportional hazards regression. All statistical analyses were performed using statistical software SAS (SAS Institute).

## Results

### Study population

A total of 191 patients were enrolled. The mean age was 74.1 ± 8.0 years, 62.8% were male, with 70.2% in NYHA Class III/IV. Patients were at high risk for cardiac surgery: Society of Thoracic Surgery Predicted Risk of Mortality (STS-PROM) score for mitral valve replacement was 7.7 ± 6.6% and European System for Cardiac Operative Risk Evaluation (EuroSCORE) II was 6.6 ± 5.3% (*Table 1*). Most patients had significant comorbidities and risk factors, including cardiac arrhythmia (70.7%), congestive heart failure (77.0%), coronary artery disease (68.1%), renal insufficiency (58.1%), and implanted pacemaker/defibrillator (40.3%). The majority (88.5%) had secondary MR, and the baseline LVEF was 45.2 ± 8.9%. Baseline severe MR was characterized by two-dimensional mitral valve effective regurgitant orifice area (EROA) 0.26 ± 0.10 cm^2^, regurgitant volume 40.9 ± 12.4 ml, and regurgitant fraction 46.4 ± 10.8%. The mean mitral valve gradient was 2.8 ± 1.3 mmHg. Tricuspid regurgitation (TR) was present in 82.5% of the patients (51.8% with TR grade ≥ 2+, 17.5% with TR grade 3+/4+).

**Table 1:** Baseline characteristics

Characteristic	Treated population (*N* = 191)
*Demographics*	
Age (years)	74.1 ± 8.0 (191)
Gender: Male	62.8% (120/191)
BMI (kg/m^2^)	27.0 ± 5.9 (191)
*Functional assessment and risk scores*	
NYHA functional class	
Class I	0.0% (0/191)
Class II	29.8% (57/191)
Class III	64.4% (123/191)
Class IV	5.8% (11/191)
STS-PROM for MV replacement (%)	7.7 ± 6.6 (191)
EuroSCORE II (%)	6.6 ± 5.3 (175)
*Baseline echocardiography*	
MR aetiology	
Primary	11.5% (22/191)
Secondary	88.5% (169/191)
Mean gradient (mmHg)	2.8 ± 1.3 (140)
EROA (cm^2^)	0.26 ± 0.10 (117)
Regurgitant volume (ml)	40.9 ± 12.4 (122)
Regurgitant fraction (%)	46.4 ± 10.8 (64)
LVEF (%)	45.2 ± 8.9 (182)
LVED dimension (cm)	5.9 ± 0.7 (184)
LVES dimension (cm)	4.8 ± 0.8 (182)
*Medical history*	
Cardiac arrhythmia	70.7% (135/191)
Congestive heart failure	77.0% (147/191)
Coronary artery disease	68.1% (130/191)
Diabetes	27.7% (53/191)
HF hospitalization in prior 6 months	44.5% (85/191)
Implanted pacemaker and/or defibrillator	40.3% (77/191)
Myocardial infarction	45.5% (87/191)
Pulmonary hypertension	51.1% (90/176)
Renal insufficiency (GFR <60 ml/min/1.73 m^2^)	58.1% (111/191)
Valvular heart disease other than mitral	35.1% (67/191)
Stroke	8.4% (16/191)

BMI, body mass index; EuroSCORE, European System for Cardiac Operative Risk Evaluation; EROA, effective regurgitant orifice area; GFR, glomerular filtration rate; HF, heart failure; LVED, left ventricular end-diastolic; LVEF, left ventricular ejection fraction; LVES, left ventricular end-systolic; MR, mitral regurgitation; MV, mitral valve; NYHA, New York Heart Association; STS-PROM, Society of Thoracic Surgeons Predicted Risk of Mortality.

### Performance and safety outcomes

**Figure 1:**
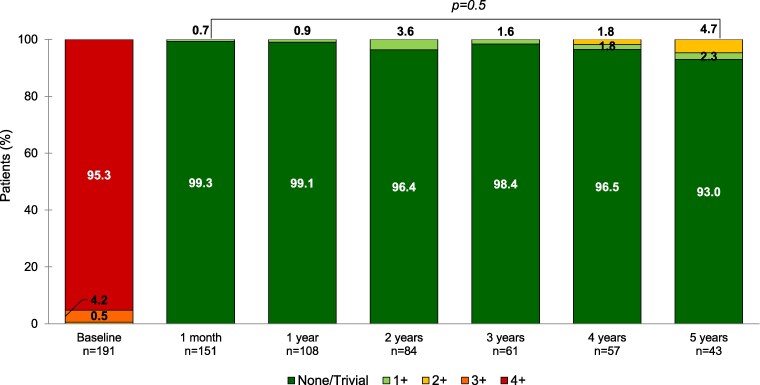
Mitral regurgitation severity through 5 years

**Figure 2:**
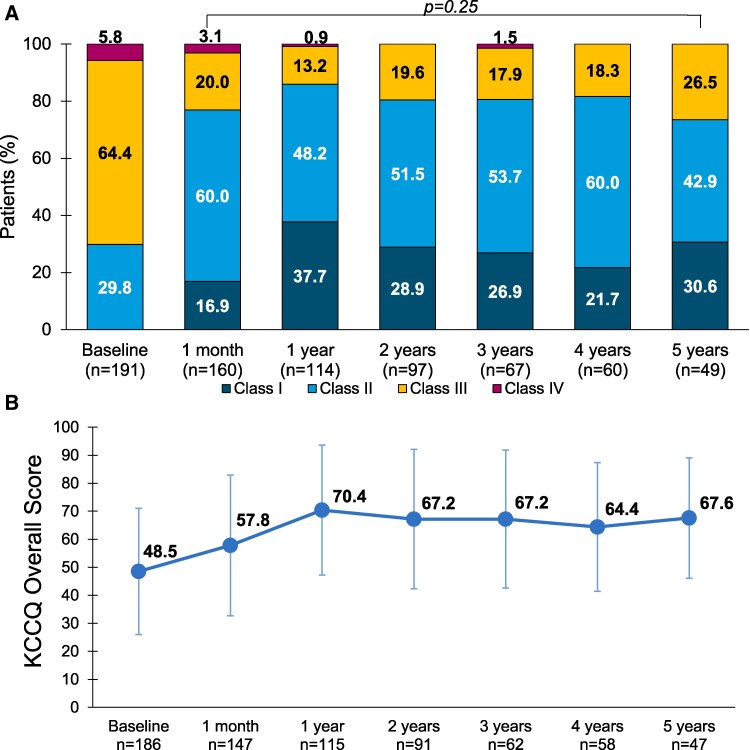
(*A*) NYHA functional class and (*B*) Kansas City Cardiomyopathy Questionnaire (KCCQ) overall score through 5 years

*Figure 1* shows MR severity through 5 years. At baseline, 99.5% of the patients had MR ≥3+; at 30 days, all alive patients had MR ≤1+. The rates of MR reduction to 1+ or less were maintained throughout the study: 98.3% at 4 years and 95.3% at 5 years (*P* = .50 for 30 days vs 5 years). At baseline, 70.2% were in NYHA Class III/IV (*Figure 2A*). The proportion of patients in NYHA Class I/II was 76.9% at 30 days and remained higher compared to baseline throughout the study (81.7% and 73.5% at 4 and 5 years, respectively; *P* = .25 for 30 days vs 5 years). KCCQ-OS score (*Figure 2B*) increased from 48.5 ± 22.5 points at baseline to 57.8 ± 25.1 points at 30 days and was 67.6 ± 21.5 points through 5 years.

**Figure 3:**
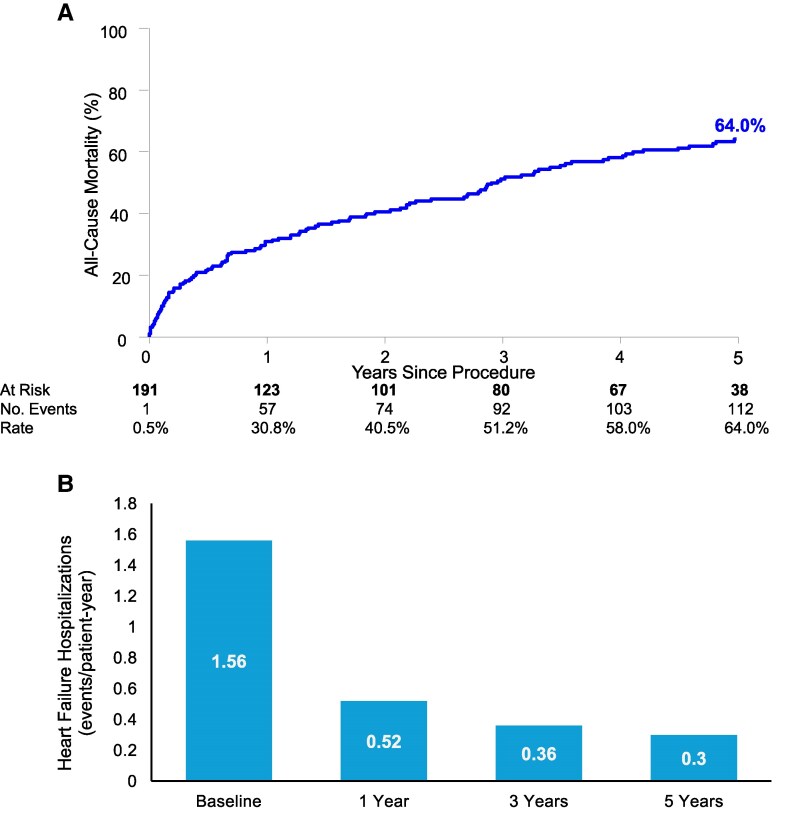
(*A*) Kaplan–Meier estimate of all-cause mortality through 5 years and (*B*) annualized heart failure hospitalization (HFH) rates before and after Tendyne TMVR (total number of HFH events divided by total follow-up years through 5 years)

The Kaplan-Meier estimate for all-cause mortality was 64.0% at 5 years (*Figure 3A*). There was no difference in all-cause mortality between primary MR (60.4%) and secondary MR (64.5%) at 5 years (*P* = .83). The annualized rate of HFH 5-years post-procedure was 0.30 (95% CI: 0.26, 0.36) events per patient-year compared with 1.56 (95% CI: 1.21, 2.00) events per patient-year at baseline (*P*  *<* .0001; *Figure 3B*). *Table 2* shows serious adverse events as annualized rates (events/patient-year). The most prevalent included: cumulative HFH (1.03 for 30 days, 0.28 for 5 years; *P* < .0001 for 30 days vs 31 days-5 years), renal insufficiency/failure (1.5 for 30 days, 0.11 for 5 years; *P* < .0001 for 30 days vs 31 days–5 years), and new atrial fibrillation (1.03 for 30 days, 0.03 for 5 years; *P* < .0001 for 30 days vs 31 days–5 years). As shown in *Table 2*, annualized rates of serious adverse events at 30 days were significantly higher than at 31 days–5 years. No structural valve degeneration events (i.e. erosion, leaflet tear, or damage to device) were observed throughout the study, but infective endocarditis occurred in 7.3% of patients by 5 years. Of the 14 patients with endocarditis, three died soon after diagnosis, one had successful re-intervention for valve replacement, and the remaining 11 patients were successfully treated with medication. Device thrombosis occurred in 5.8% of patients by 5 years (all patients remained on anti-coagulant medication during the study). Five of these 11 patients were anticoagulated at baseline. A total of seven mitral valve re-interventions (3.7% at 5 years) were performed during the study for paravalvular leak (PVL) (*n* = 5), device malposition (*n* = 1), and device thrombosis (*n* = 1). There were four cases of pad repositioning (tether length adjustment), which occurred between 10- and 37-day post-procedure, to successfully treat PVL (*n* = 3) and migration (*n* = 1).

**Table 2:** Serious adverse events

Serious adverse event	Annualized rates: event/patient-year (number of events)		*P* value (for 0–30 days vs 31 days–5 years)^1^
	0–30 days	31 days –5 years	
Total patient-years of follow-up	14.6	474.3	NA
Heart failure hospitalization	1.03 (15)	0.28 (132)	**<**.**0001**
Bleeding			
Life-threatening	0.62 (9)	0.03 (13)	**<**.**0001**
Fatal	0.07 (1)	0.01 (4)	.06
Renal insufficiency/failure	1.50 (22)	0.11 (54)	**<**.**0001**
New atrial fibrillation	1.03 (15)	0.03 (15)	**<**.**0001**
Haemolysis	0.07 (1)	0.004 (2)	.**02**
Device thrombosis	0.21 (3)	0.02 (8)	.**0003**
Endocarditis	0.21 (3)	0.03 (12)	.**01**
Structural valve degeneration^a^	0.000 (0)	0.000 (0)	NA
Device migration	0.000 (0)	0.000 (0)	NA
Device embolism	0.000 (0)	0.000 (0)	NA

The bold *P* values are statistically significant.

^a^Defined as device-specific adverse events (erosion, tear, or damage to device).

### Additional echocardiographic assessments

*Table 3* describes additional echocardiographic assessments. Forward stroke volume increased from 50 ± 15 ml at baseline to 57 ± 24 ml at 5 years (*P* = .048). The mean MV gradient was 2.8 ± 1.3 mmHg at baseline compared with 3.5 ± 2.0 mmHg at 5 years (*P* = .054), and the mean MV area decreased from 4.2 ± 1.0 cm^2^ at baseline to 3.7 ± 0.8 cm^2^ at 5 years (*P* < .0001). The LV outflow tract (LVOT) mean gradient did not change significantly, from 1.4 ± 0.7 mmHg at baseline to 1.6 ± 0.8 mmHg at 5 years (*P* = .23). No PVL was observed at 5 years.

**Table 3:** Echocardiography results at 5 years—paired analysis

Parameter (*n*)	Baseline	5 years	Δ 5Y—baseline	*P*-value
***Global heart function***				
** Forward stroke volume (m**l**)**	50.4 ± 19.6 (28)	59.3 ± 24.9 (28)	8.9	.048
** Cardiac output (L/min)**	3.6 ± 1.1 (26)	4.2 ± 1.8 (26)	0.6	.109
***Mitral valve***				
** MV mean gradient (mmHg)**	3.1 ± 1.4 (27)	3.7 ± 2.3 (27)	0.6	.054
** MV area (cm^2^)**	4.3 ± 1.1 (34)	3.7 ± 0.9 (34)	−0.6	<.0001
** PVL grade**				
** None**	100 (38)^a^	100 (38)	—	—
** 1+**	0.0 (38)^a^	0.0 (38)	—	—
** 2+**	0.0 (38)^a^	0.0 (38)	—	—
** 3+**	0.0 (38)^a^	0.0 (38)	—	—
** 4+**	0.0 (38)^a^	0.0 (38)	—	—
***Left ventricle (LV)***				
** LVED dimension (cm)**	5.9 ± 0.7 (43)	6.2 ± 0.9 (43)	0.3	.004
** LVED volume (ml)**	169.6 ± 61.6 (30)	157.2 ± 71.0 (30)	−12.4	.071
** LVEDV indexed (ml/m^2^)**	87.8 ± 26.5 (30)	80.7 ± 30.2 (30)	−7.1	.036
** LVEF (%)**	45.3 ± 8.9 (40)	36.9 ± 12.8 (40)	−8.5	<.0001
** LVOT mean gradient (mmHg)**	1.5 ± 0.7 (31)	1.7 ± 0.8 (31)	0.2	.225
***Right ventricle (RV)***				
** RV systolic pressure (mmHg)**	47.3 ± 15.1 (19)	42.7 ± 13.9 (19)	−4.6	.272
***Tricuspid valve***				
** Tricuspid regurgitation (TR) grade (%)**				.249^b^
** None or trivial**	8.3 (36)	0.0 (36)	—	—
** 1+**	33.3 (36)	61.1 (36)	—	—
** 2+**	38.9 (36)	36.1 (36)	—	—
** 3+**	19.4 (36)	2.8 (36)	—	—
** 4+**	0.0 (36)	0.0 (36)	—	—

Least Square Means estimate using repeated measure mixed model and adjusting for baseline measure.

CI, confidence interval; LVED, left ventricular end-diastolic; LVES, left ventricular end-systolic; LVEF, left ventricular ejection fraction; LVOT, left ventricular outflow tract; MV, mitral valve; PVL, paravalvular leak; NA, not applicable.

^a^For PVL grade only, pre-discharge data is shown instead of baseline.

^b^Bowker’s test.

LV end-diastolic dimension (LVEDD) increased from 5.9 ± 0.7 cm at baseline to 6.2 ± 0.9 cm at 5 years (*P* = .004) and LV end-systolic dimension (LVESD) increased from 4.8 ± 0.8 cm at baseline to 5.1 ± 1.3 cm at 5 years (*P* = .035); however, indexed LV end-diastolic volume (LVEDVi) decreased from 87.8 ± 26.5 ml/m^2^ at baseline to 80.7 ± 30.2 ml/m^2^ at 5 years (*P* = .036). There was a significant decrease in LVEF (from 45% ± 9% at baseline to 37% ± 13% at 5 years; *P* < .0001). Right ventricular (RV) systolic pressure remained elevated: from 45 ± 14 mmHg at baseline to 38 ± 13 mmHg at 5 years (*P* = .27). Tricuspid regurgitation (TR) ≥3+ was 19.4% at baseline and 2.8% at 5 years (*P* = .25).

### Medications follow-up in deceased vs surviving patients

*Table 4* shows a comparison of the cardiovascular medications taken by the deceased vs surviving patients at baseline and at their last follow-up (all implanted patients with at least a 3-month visit were included). Cardiovascular medications included anticoagulant, antiplatelet, calcium channel blocker, inotropes, and statins. At baseline, significantly more patients alive at 5-year follow-up were on ACE inhibitors (50.0%) vs patients who were deceased (26.6%, *P* = .003). At the last follow-up, surviving patients had a significantly higher proportion of four different heart failure medications: ARBs (40.0% of the surviving patients vs 16.5% of the deceased patients, *P* = .001), diuretics (97.1% of the surviving patients vs 84.8% of the deceased patients, *P* = .01), vasodilators (24.3% of the surviving patients vs 11.4% of the deceased patients, *P* = .039), and SGLT2 inhibitors (12.9% of the surviving patients vs 2.5% of the deceased patients, *P* = .016). No other statistical differences were observed in any of the other medications.

**Table 4:** Medications follow-up in deceased vs surviving patients

Medications	Deceased (*n* = 79)	Surviving (*n* = 70)	Difference	*P* value*
*Baseline*				
ACE inhibitor	26.6% (21/79)	50.0% (35/70)	−23.4%	.**003**
Angiotensin receptor blocker (ARB)	22.8% (18/79)	22.9% (16/70)	−0.1%	.992
Angiotensin receptor–neprilysin inhibitor (ARNI)	3.8% (3/79)	2.9% (2/70)	0.9%	1.000
Anticoagulant	51.9% (41/79)	55.7% (39/70)	−3.8%	.641
Antiplatelet (including aspirin)	57.0% (45/79)	62.9% (44/70)	−5.9%	.464
Beta blocker	87.3% (69/79)	87.1% (61/70)	0.2%	.971
SGLT2 inhibitor	0.0% (0/79)	0.0% (0/79)	0.0%	1.000
Calcium channel blocker	10.1% (8/79)	7.1% (5/70)	3.0%	.520
Digitalis	3.8% (3/79)	10.0% (7/70)	−6.2%	.191
Diuretic	84.8% (67/79)	91.4% (64/70)	−6.6%	.216
Inotropes	1.3% (1/79)	0.0% (0/70)	1.3%	1.000
Statin	64.6% (51/79)	70.0% (49/70)	−5.4%	.480
Vasodilators	16.5% (13/79)	14.3% (10/70)	2.17%	.714
*Last follow-up*				
ACE inhibitor	26.6% (21/79)	28.6% (20/70)	−2.0%	.786
Angiotensin receptor blocker (ARB)	16.5% (13/79)	40.0% (28/70)	−23.5%	.**001**
Angiotensin receptor–neprilysin inhibitor (ARNI)	2.5% (2/79)	5.7% (4/70)	−3.2%	.420
Anticoagulant	81.0% (64/79)	90.0% (63/70)	−9.0%	.123
Antiplatelet (including aspirin)	46.8% (37/79)	40.0% (28/70)	6.8%	.401
Beta blocker	81.0% (64/79)	80.0% (56/70)	1.0%	.876
SGLT2 inhibitor	2.5% (2/79)	12.9% (9/70)	−10.3%	.**016**
Calcium channel blocker	5.1% (4/79)	4.3% (3/70)	0.8%	1.000
Digitalis	8.9% (7/79)	4.3% (3/70)	4.6%	.336
Diuretic	84.8% (67/79)	97.1% (68/70)	−12.3%	.**010**
Inotropes	7.6% (6/79)	1.4% (1/70)	6.2%	.121
Statin	60.8% (48/79)	70.0% (49/70)	−9.2%	.238
Vasodilators	11.4% (9/79)	24.3% (17/70)	−12.9%	.**039**

The bold *P* values are statistically significant.

^*^From Chi-square test, and from Fisher's exact test when Cochran's rule is not met.

### Predictors of all-cause mortality and HFH within 5 years

*Table 5* shows the results of univariable and multivariable Cox regression analysis of all-cause mortality and HFH within 5 years. Parameters associated with a significantly increased risk of all-cause mortality at 5 years by univariable regression included: baseline NYHA Class III/IV, shorter 6-min walk test, history of hypertension, renal insufficiency, elevated NT pro-BNP, tricuspid regurgitation (TR) >2+ at discharge or 30 days, and higher right atrial pressure at discharge or 30 days. Factors associated with a significantly increased risk of HFH by univariable regression included history of hypertension, renal insufficiency and smaller EROA. By multivariable regression, history of hypertension was associated with increased risk of all-cause mortality or HFH at 5 years and renal insufficiency was associated with increased risk of HFH at 5 years.

**Table 5:** Predictors of all-cause mortality and heart failure hospitalization (HFH) within 5 years by univariable and multivariable cox regression

Parameter	Coefficient	Hazard ratio [95% CI]	*P* value*
*Univariable—All-cause mortality*			
NYHA functional class III/IV	0.46	1.58 [1.02, 2.46]	.041
Six-min walk test (10 m)	−0.02	0.98 [0.97, 1.00]	.025
History of hypertension	0.63	1.87 [1.13, 3.11]	.015
Renal insufficiency (<60 ml/min/1.73^2^)	0.51	1.66 [1.12, 2.45]	.012
HF hospitalization within past 6 months	0.09	1.09 [0.75, 1.58]	.650
Elevated NT pro-BNP (>1600 pg/ml)	0.50	1.65 [1.08, 2.52]	.020
RVSP (5 mmHg) at baseline	−0.12	0.89 [0.78, 1.01]	.071
RVSP (5 mmHg) at discharge or 30 days	0.07	1.07 [0.97, 1.19]	.150
RAP (mmHg) at baseline	0.03	1.03 [0.97, 1.10]	.306
RAP (mmHg) at discharge or 30 days	0.07	1.08 [1.02, 1.13]	.005
TR ≥2+ at baseline	0.08	1.08 [0.73, 1.61]	.699
TR ≥2+ at discharge or 30 days	0.45	1.56 [1.03, 2.38]	.038
*Univariable—heart failure hospitalization (HFH)*			
History of hypertension	0.87	2.39 [1.26, 4.55]	.008
Renal insufficiency (<60 ml/min/1.73^2^)	0.71	2.03 [1.24, 3.32]	.005
HF hospitalization within past 6 months	0.12	1.13 [0.72, 1.78]	.594
Elevated NT pro-BNP (>1600 pg/ml)	0.45	1.56 [0.95, 2.57]	.080
EROA (10 mm^2^)	−0.51	0.60 [0.40, 0.89]	.010
RVSP (5 mmHg) at baseline	−0.12	0.88 [0.77, 1.01]	.068
RVSP (5 mmHg) at discharge or 30 days	0.04	1.04 [0.93, 1.16]	.483
RAP (mmHg) at baseline	−0.01	0.99 [0.92, 1.06]	.750
RAP (mmHg) at discharge or 30 days	0.04	1.04 [0.99, 1.10]	.128
TR ≥2+ at baseline	0.03	1.03 [0.63, 1.69]	.910
TR ≥2+ at discharge or 30 days	0.36	1.44 [0.89, 2.32]	.136
*Multivariable—all-cause mortality or HFH*			
History of hypertension	0.62	1.85 [1.16, 2.95]	.010
*Multivariable—HFH*			
Renal insufficiency (<60 ml/min/1.73^2^)	0.69	1.99 [1.20, 3.29]	.008

CI, confidence interval; EROA, effective regurgitant orifice area; HFH, hear failure hospitalization; TR, tricuspid regurgitation; RVSP, right ventricular systolic pressure; RAP, right atrial pressure.

^*^From Wald chi-square test.

## Discussion

Transcatheter options for the treatment of mitral regurgitation in patients who are ineligible for mitral valve surgery or TEER are needed. This report comprises the complete cohort of patients enrolled in the prospective Tendyne Expanded Clinical Study through 5-year follow-up, extending previously published 3-year data^14^ on clinical outcomes following TMVR with the Tendyne system. At 5 years, treatment with Tendyne demonstrated sustained durability of previously reported clinical benefits, including (i) consistent elimination of severe MR (grade > 2+), (ii) preservation of functional capacity, (iii) improvement in quality of life as reflected by stable KCCQ-OS score, (iv) persistently low rates of serious adverse events throughout the 5-year follow-up period, (v) reduced need for heart failure hospitalization, and (vi) sustained haemodynamic benefits beyond the elimination of severe MR.

The 5-year all-cause mortality observed in this cohort (64%) reflects the advanced clinical profile of patients selected for TMVR with the Tendyne system. These individuals were characterized by high or prohibitive surgical risk, as determined by multidisciplinary heart teams’ evaluation, and carried a substantial burden of comorbidities including advanced heart failure, renal dysfunction, and frailty. Three deaths (1.6%) observed were associated with valve complications: device thrombosis (*n* = 2) and re-intervention for malposition (*n* = 1). The device thrombosis-related deaths occurred at approximately 2- and 3-year post-procedure (782- and 1029-day post-procedure). The death event associated with device malposition occurred 78 days post-procedure. Despite the sobering nature of this outcome, the all-cause mortality rate is consistent with 5-year rates reported for other transcatheter valve therapies in similar patient populations.^15–17^ The association between baseline NYHA Class III/IV, renal disease, hypertension, and tricuspid regurgitation and an increased risk of all-cause mortality, as observed in this study, was also reported in the COAPT,^18^ EVEREST II^19^ and MITRAL trials.^17^ These findings underscore the importance of patient selection and risk stratification in interpreting long-term outcomes in this complex population.

Guideline-directed medical therapy (GDMT) did not became widely used in TMVR patients until 2018 to 2020, when TMVR clinical trials began requiring the same maximally tolerated GDMT used in TEER trials. Therefore, the incidence of heart failure medication for patients enrolled in the Tendyne Expanded Clinical Study at baseline was relatively low. It is possible that outcomes following Tendyne TMVR could have been improved if patients had received current GDMT or if they had been medically optimized more stringently during the 5-year follow-up. It should also be noted that the study was performed prior to the widespread use of SGLT-2 inhibitors, which have been shown to have a favourable prognostic benefit in this patient population.^20,21^

Echocardiography results reported for this study were similar to or improved compared to those reported in the 5-year follow-ups for COAPT,^16^ and MITRAL trials.^17^ MR grade ≤2+ at 5 years was 100% in the present study (vs 94.7% in the COAPT device group^16^ and 96% in the MITRAL trial^20^), whereas decreases in LVEF and RV systolic pressure were similar to those reported in the COAPT device group and the MITRAL trial at 5 years.^16,17^ Of note, the decrease in LVEF observed in this study (from 45% at baseline to 37% at 5 years) is similar to the observed decreases in the COAPT device group^16^ (from 31% at baseline to 28% at 5 years), MITRAL trial Valve-in-Valve arm^17^ (from 56% at baseline to 48% at 5 years), and in the Intrepid TMVR global pilot study^15^ (from 44% at baseline to 39% at 5 years). Key parameters remained favourable over the 5-year follow-up period, including stable LV dimensions, a reduction in tricuspid regurgitation and RV systolic pressure, and consistent MV gradient and MV area. LVOT gradients remained unchanged.

Importantly, at 5 years, there were no instances of structural valve degeneration (erosion, tear, or damage to device), and the endocarditis rate was 7.3%, which compares favourably to the corresponding rates reported in surgical mitral valve replacement studies (e.g. 1.3% and 20.7%, respectively, for the SJM Epic valve,^22^ 7.8% endocarditis for the On-X mitral valve^23^). Overall, serious adverse events following TMVR increased progressively over the first 3 years of the study, and then mostly stabilized by years 4 and 5.

### Limitations

There were several limitations for this study. First, this investigation was conducted as a single-arm interventional study, and the absence of a control group restricts the ability to draw comparative conclusions against other MR therapies. Second, clinical and echocardiographic follow-up was only available for surviving patients. Lastly, perioperative management and long-term medical therapy for this complex patient population were not standardized within the study protocol, which may have influenced clinical outcomes, including the observed mortality rates. Optimal periprocedural outcomes typically require rigorous and comprehensive medical management, ideally involving input from heart failure specialists. It is plausible that longer-term outcomes could have been enhanced through systematic optimization of GDMT.

## Conclusion

Treatment with the Tendyne Mitral Valve System was associated with long-term sustained reduction in MR severity, symptomatic improvement, and no instances of SVD in a high-risk cohort over 5 years. These findings support TMVR with Tendyne as a potential alternative for patients who are unsuitable for mitral valve surgery or TEER.

## Declarations

### Data Availability

No data were generated or analysed for this manuscript.

### Pre-registered Clinical Trial Number

None supplied.
